# Supplementary material for: Age-Dependent and Aβ-Induced Dynamic Changes in the Subcellular Localization of HMGB1 in Neurons and Microglia in the Brains of an Animal Model of Alzheimer’s Disease
Source: Cells. 2024 Jan 18;13(2):189. doi: 10.3390/cells13020189 (PMC10814163; doi:10.3390/cells13020189)
Supplement: Supplementary file 1 [file cells-13-00189-s001.zip › cells-2660558-supplementary.pdf]

Supplementary materials

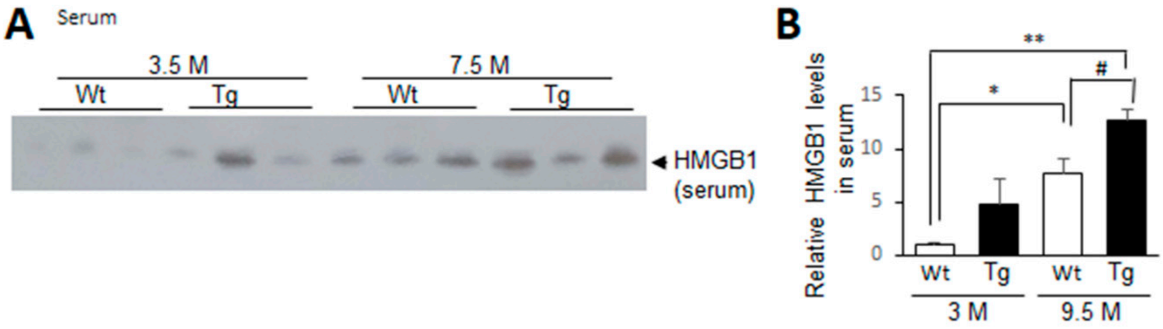

Supplementary Figure S1. HMGB1 protein levels in the serum of 3.5 M and 7.5 M Tg-APP/PS1 and age-matched control mice

Protein samples were prepared from the sera of Tg-APP/PS1 or age-matched Wt mice at 3.5 or 7.5 M of age, and HMGB1 protein levels were determined by immunoblotting. Representative immunoblots are presented in A, and quantification results are presented as means ± SEMs (n=3 for 3.5 M and n=5 for 9.5 M) in B. *p < 0.05, **p < 0.01 vs. 3.5 M Wt, $^{\#}$p < 0.05 between indicated groups.

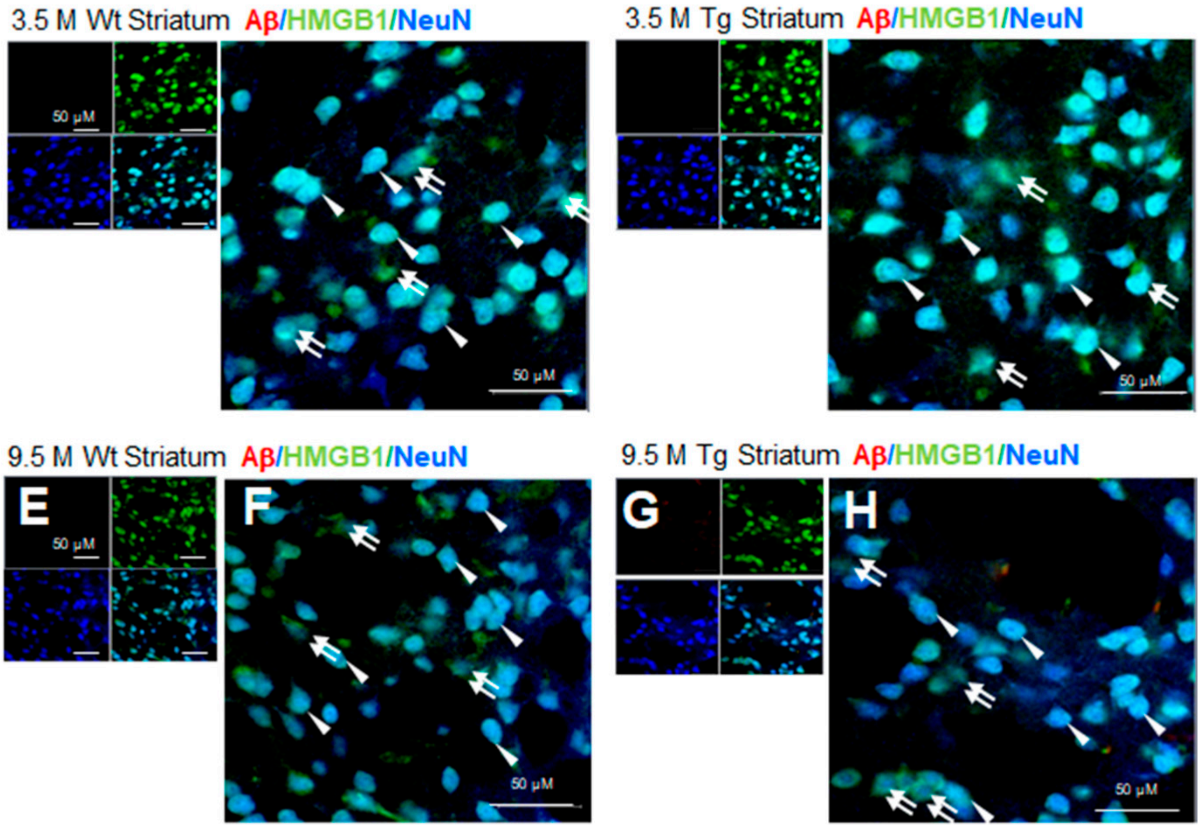

Supplementary Figure S2. Cellular and subcellular localization of HMGB1 in striata of 3.5 and 9.5 M Tg-APP/PS1 and age-matched control mice

Coronal brain sections were prepared from 3.5 M (C and D) or 9.5 M Tg (G and H) and age-matched control (A, B, E, and F) mice. Triple immunofluorescence staining was performed using anti-Ab, anti-HMGB1, and anti-NeuN (A-H) antibodies. Arrowheads indicate the immunoreactivity of anti-HMGB1 in neurons (B, D, F, and H). Double arrows indicate immunoreactivity of anti-HMGB1 in NeuN-negative cells (B, D, F, and H). Scale bars represent 20 or 50 μm.
